# Will the introduction of β-lactamase inhibitors alter the adverse event profile of parent penicillins antibiotics? a pharmacovigilance study based on amoxicillin-clavulanate and ampicillin-sulbactam

**DOI:** 10.3389/fphar.2026.1772354

**Published:** 2026-03-13

**Authors:** Yanlang He, Yunxia Liao, Guie Xiao, Dinghui Zhou, Liuyun Huang, Sheng Wei

**Affiliations:** 1 Department of Infectious Disease, Shaoyang Central Hospital, Shaoyang, China; 2 Department of General Medicine, the Second Affiliated Hospital of Wannan Medical College, Wuhu, China

**Keywords:** adverse event, amoxicillin-clavulanate, ampicillin-sulbactam, FAERS, β-lactamase inhibitors

## Abstract

**Objective:**

This study aimed to investigate whether the introduction of **β**-lactamase inhibitors (BLIs) alters the adverse event (AE) profiles of parent penicillin antibiotics, using real-world pharmacovigilance data.

**Methods:**

A disproportionality analysis was conducted using reports from the FDA Adverse Event Reporting System database (2004–2024). The research compared the safety profiles of amoxicillin versus amoxicillin-clavulanate (ACP) and ampicillin versus ampicillin-sulbactam (ASS) by employing multiple statistical methods, including the Reporting Odds Ratio (ROR), Proportional Reporting Ratio (PRR), Bayesian Confidence Propagation Neural Network (BCPNN), and Empirical Bayesian Geometric Mean (EBGM). The signals were considered significant only when meeting multiple algorithm criteria (Reporting Odds Ratio, Proportional Reporting Ratio, Bayesian Confidence Propagation Neural Network, Empirical Bayesian Geometric Mean). Subgroup analyses by age and gender were performed.

**Results:**

Analysis of over 200,000 reports revealed distinct AE spectra. ACP demonstrated a significantly stronger association with hepatobiliary disorders (e.g., drug-induced liver injury, ROR 35.41) compared to amoxicillin alone (ROR 4.98). In contrast, amoxicillin monotherapy showed stronger signals for certain immune-mediated skin reactions. For ampicillin, monotherapy was strongly linked to pregnancy/perinatal events, whereas ASS showed higher risks for gastrointestinal disorders (e.g., hemorrhagic enterocolitis), specific skin reactions, and allergic events. Subgroup analyses indicated that hepatobiliary and renal risks with ACP were more prominent in males and the elderly (≥60 years), while severe skin reactions with ampicillin were more frequent in females.

**Conclusion:**

The addition of BLIs significantly may modify the AE profiles of parent penicillins, introducing distinct organ-specific risks. These findings underscore the necessity for personalized risk assessment and monitoring in clinical practice, tailored to the specific combination agent and patient demographics.

## 1 Introduction

The discovery and application of penicillin represent a landmark achievement in the history of human medicine. Since its accidental discovery in 1928 by British bacteriologist Alexander Fleming, penicillin antibiotics—including both natural and semisynthetic penicillins—have substantially reduced mortality from a wide range of bacterial infections and have saved hundreds of millions of lives. Currently, penicillin antibiotics remain the most widely used antibiotics globally across various clinical settings, due to their high safety profile, low cost, broad antibacterial spectrum, and multiple dosage forms. However, the extensive and often inappropriate use of penicillins has led to the rapid emergence of bacterial resistance (Goossens et al., 2005). This includes unnecessary prescribing for self-limiting or likely viral syndromes, empiric escalation to broader regimens without clear microbiological indication, and suboptimal dosing or premature discontinuation (Havey et al., 2015; Valladales-Restrepo et al., 2025), which partly contribute to the emergence of resistant bacteria such as β-lactamase-producing *Haemophilus* influenzae, *Moraxella catarrhalis*, and methicillin-resistant *Staphylococcus aureus* (MRSA) (File et al., 2004; Geisinger and Isberg, 2017).

Mechanistically, the expression of β-lactamase enzymes produced by pathogens, which hydrolyze the amide bond of the β-lactam ring, is the major mechanism for bacterial resistance to β-lactams (Kang et al., 2024). Consequently, β-lactamase inhibitors have been developed. These agents are β-lactam-based molecules structurally related to penicillins. They act as “suicide substrates” by forming covalent acyl-enzyme complexes with serine β-lactamases, thereby enhancing therapeutic efficacy and reducing the development of resistance (Bush and Bradford, 2016). A series of clinical studies have confirmed the effectiveness of such combination regimens in treating infections caused by β-lactamase-producing resistant bacteria (Hirai et al., 2022; Xu et al., 2021; Wilkowski et al., 2023). Currently, six β-lactamase inhibitors (BLIs)—avibactam, clavulanic acid, relebactam, sulbactam, tazobactam, and vaborbactam—are approved by the United States Food and Drug Administration (USFDA). Prior evidence suggests that common adverse event (AEs) across BLIs included hematologic disorders, hypersensitivity reactions, emergent infections, organ dysfunction, and neurological complications. Signal detection revealed specific associations: septic shock and respiratory failure with avibactam; lymphadenopathy and congenital anomalies with clavulanic acid; antimicrobial resistance and epilepsy with relebactam; disseminated intravascular coagulation and cardiac arrest with sulbactam; and agranulocytosis and conduction abnormalities with tazobactam (Sridharan and Sivaramakrishnan, 2025).

However, whether the introduction of BLIs alter the AEs profile of parent penicillins antibiotics remains controversial. For example, amoxicillin (used alone or in combination with clavulanate) remains one of the most extensively prescribed semisynthetic penicillins. Although they are often regarded as “twin drugs,” differences exist in their antibacterial activity and safety profiles. Substantial evidence suggests that the clavulanate component itself may trigger adverse reactions, thereby exposing patients to additional and potentially unnecessary risks (Salvo et al., 2009). In children with acute sinusitis treated in outpatient settings, amoxicillin-clavulanate (ACP) was associated with a slightly higher risk of treatment failure (due to insufficient efficacy or intolerance) compared to amoxicillin alone, along with an increased incidence of gastrointestinal symptoms and *Candida* albicans infections (Savage et al., 2023). Furthermore, a 4-year pharmacovigilance analysis indicated that amoxicillin/clavulanate was more strongly associated with hepatotoxicity risk and increased antibiotic resistance compared to amoxicillin alone (White et al., 2004). Additionally, an earlier Italian study suggested that amoxicillin/clavulanate appeared to be linked to a higher risk of Stevens-Johnson syndrome, purpura, and hepatitis than amoxicillin monotherapy. Notably, the reported rate of hepatitis was on average nine times higher for amoxicillin/clavulanate than for amoxicillin alone (Salvo et al., 2007). However, most of these studies are limited by small sample sizes, short observation periods, homogeneous patient populations, and being single-drug or single-center investigations, raising questions about the generalizability and clinical applicability of their findings. Moreover, for other semisynthetic penicillin-BLI combinations such as ampicillin-sulbactam (ASS) and piperacillin-tazobactam, whether similar increases in adverse reactions occur compared to their parent antibiotics remains unstudied. Consequently, current understanding in this area remains limited, and many clinicians lack a clear conceptual distinction, often conflating the adverse reaction profiles of single antibiotics and combination agents when prescribing.

Given the continued widespread clinical use of semisynthetic penicillins and their combination formulations, clarifying whether significant differences exist in their respective AE profiles is crucial for clinicians to make appropriate drug choices and minimize adverse outcomes. Therefore, this study aims to utilize drug adverse event reports from the US Food and Drug Administration Adverse Event Reporting System (FAERS) database. By comparing the safety profiles of two commonly used semisynthetic penicillins and their corresponding combination agents (amoxicillin vs. ACP, ampicillin vs. ASS), we intend to conduct a preliminary investigation into whether the introduction of β-lactamase inhibitors alters the adverse event characteristics of the parent antibiotics. The findings are expected to provide a reference for future mechanistic studies on this topic.

## 2 Methods

### 2.1 Data source

FAERS is an online database maintained by the FDA. Since its launch in 2004, the system has collected reports of adverse drug reactions, medication errors, and product quality complaints from healthcare professionals, manufacturers, and patients in over 150 countries and regions, presented in the form of individual case safety reports (He et al., 2025). Containing data from diverse populations and clinical settings, FAERS enables global surveillance of AEs associated with drugs and medical products, making it a crucial tool for identifying potential safety risks of marketed medications (Tang et al., 2025).

The FAERS system employs the latest version of the Medical Dictionary for Regulatory Activities (MedDRA®) to accurately code each adverse drug reaction (ADR), and uses the World Health Organization’s Anatomical Therapeutic Chemical (ATC) Classification System to standardize drug nomenclature. The FAERS database consists of eight key data tables: Patient Demographic and Administrative Information (DEMO), Drug Information (DRUG), Adverse Reaction Records (REAC), Patient Outcomes (OUTC), Report Sources (RPSR), Therapy Timeline (THER), Indications for Use or Diagnosis (INDI), and Deleted Case Records (DELETED). As such, it provides detailed information on report volume, patient demographics (such as age and sex), and the severity of adverse drug events (ADEs). Each record includes variables such as “primaryid,” which serves as the unique identifier for an individual ADR report, and “caseid,” which uniquely identifies a patient case. Through these variables, specific information regarding patients and AEs can be retrieved. All data files are publicly accessible on the FDA website at https://fis.fda.gov/extensions/FPD-QDE-FAERS/FPD-QDE-FAERS.html. Since FAERS data are publicly available and accessible to anyone, neither informed consent nor approval from an institutional review board was required for this study.

### 2.2 Data processing

For this study, ASCII report files were downloaded from the FAERS database covering the period from 1 July 2004, to 30 September 2024. The year 2004 marks the inception of the FAERS database, which ensures the inclusion of the most comprehensive historical data since its establishment, thereby providing an adequate sample size for analysis. The 20-year data span helps capture long-term trends and evolution in adverse event signals, reducing bias from short-term fluctuations and reflecting the latest clinical drug safety landscape. This ensures the timeliness and practical relevance of the study findings. To address inherent limitations of spontaneous reporting systems, such as duplicate or withdrawn reports, the following cleaning steps were performed in accordance with FDA recommendations (https://www.fda.gov/drugsatfda). Duplicate reports were removed by first sorting the DEMO table by the fields PRIMARYID, CASEID, and FDA_DT (the date the FDA received the case). For entries with identical CASEIDs, the record with the latest FDA_DT was retained. If both CASEID and FDA_DT were identical, the entry with the largest PRIMARYID was kept (Xia et al., 2025). Subsequently, data from files marked for deletion in the FAERS database were extracted and removed to exclude invalid reports. Reports with chronological inconsistencies (e.g., drug administration dates later than the event onset date) or missing critical date information were also excluded. The Medical Dictionary for Regulatory Activities (MedDRA, version 25.0) organizes adverse event terminology into a five-level hierarchy: System Organ Class (SOC), High-Level Group Term (HLGT), High-Level Term (HLT), Preferred Term (PT), and Lowest Level Term (LLT). In FAERS pharmacovigilance analyses, AEs are coded using MedDRA PTs, which may encompass not only pathological reactions but also clinical statuses or outcomes documented during medication use. Each of which can be assigned to one or more SOCs. For this analysis, only PTs with an event count ≥3 were included. In FAERS, the role code assigned by the reporter indicates the suspected causal relationship of a drug to the adverse event, with categories including Primary Suspect (PS), Secondary Suspect (SS), Concomitant (C), and Interacting (I). To enhance the reliability of the findings, the present analysis was restricted to reports where the drug of interest was assigned the “Primary Suspect” (PS) role code in the DRUG table (Mascolo et al., 2021; Moore et al., 2022; Zhou et al., 2022). Finally, epidemiological baseline information was extracted from the cleaned reports, including patient age, reporting country, reporter type, report date, route of administration, patient outcomes, and the time to onset of the drug-related AEs.

### 2.3 Data analysis

This study employed disproportionality analysis to evaluate the association between drugs and AEs. This method compares the observed frequency ratios in exposed versus non-exposed populations using contingency tables, with the specific algorithms detailed in Table 1. In the present study, we calculated the Reporting Odds Ratio (ROR), Proportional Reporting Ratio (PRR), Bayesian Confidence Propagation Neural Network (BCPNN), and Empirical Bayesian Geometric Mean (EBGM). The ROR is a simple and highly sensitive method but lacks specificity and is not applicable when the number of reports for a target adverse event is less than three (Jiao et al., 2024). In contrast, the PRR offers greater specificity (Rothman et al., 2004). The EBGM aids in providing more stable estimates, particularly for rare signals with low report counts (Hunt et al., 2014). The BCPNN performs well in integrating data from multiple sources and conducting cross-validation (Bate et al., 1998). To enhance signal reliability and avoid false positives, a signal was considered relevant only if it met the detection criteria of all four algorithms simultaneously (Shu et al., 2022). Typically, these algorithms yield higher scores when the target drug is more likely to induce the target adverse event compared to all other drugs in the database. The specific formulas and thresholds for all algorithms are detailed in Table 2. Data extraction was performed using MySQL 8.0, and statistical analysis was conducted with RStudio (version 4.2.2).

**TABLE 1:** Four-grid table for signal detection.

Research object	Drug-related ADEs	Non-drug-related ADEs	Total
Drug	a	b	a+b
Non-drug	c	d	c+d
Total	a+c	b+d	N=a+b+c+d

ADE, adverse drug events. A is the number of cases where a specific adverse event occurred after using the target drug of the study, b is the number of cases where the target drug of the study were used but the specific adverse event did not occur, c is the number of cases where the specific adverse event occurred without the use of the target drug of the study, d is the number of cases where neither the target drug of the study were used nor the specific adverse event occurred.

**TABLE 2:** Four main algorithms are used to evaluate the correlation between the target drug of the study and AEs. This includes ROR, PRR, BCPNN, and EBGM methods, formulas, and thresholds.

Method	Formula	Threshold
ROR	$ROR=\left(a\;/\;c\right)/\left(b\;/\;d\right)$	a ≥3
	$95\%CI=e^{\ln\left(ROR\right)\pm 1.96\sqrt{\left(\frac{1}{a}+\frac{1}{b}+\frac{1}{c}+\frac{1}{d}\right)}\;}$	ROR ≥2 95%CI (lower limit) > 1
PRR	$\text{PRR}=a\left(c+d\right)/c/\left(a+b\right)$	a ≥3
	$95\%CI=e^{\ln\;\left(\text{PRR}\right)\pm 1.96\sqrt{\;\frac{1}{a}-\frac{1}{a+b}+\frac{1}{c}-\frac{1}{c+d}\;}}$	PRR ≥2 95% CI (lower limit) > 1
BCPNN	$IC=\log_{2}\frac{a\left(a+b+c+d\right)}{\left(a+b\right)\left(a+c\right)}$	IC025 > 0
	$\gamma =\gamma ij\frac{\left(N+\alpha \right)\left(N+\beta \right)}{\left(a+b+\alpha i\right)\left(a+c+\beta j\right)}$ $E\left(IC\right)=\log_{2}\frac{\left(a+\gamma ij\right)\left(N+\alpha \right)\left(N+\beta \right)}{\left(N+\gamma \right)\left(a+b+\alpha i\right)\left(a+c+\beta j\right)}$	
	$V\left(IC\right)=\frac{1}{{\left(\ln2\right)}^{2}}\left[\frac{\left(a+b+c+d\right)-a}{a\left(1+a+b+c+d\right)}+\frac{\left(a+b+c+d\right)-\left(a+b\right)}{\left(a+b\right)\left(1+a+b+c+d\right)}+\frac{\left(a+b+c+d\right)-\left(a+c\right)}{\left(a+c\right)\left(1+a+b+c+d\right)}\right]$	
	$IC025=E\left(IC\right)-2\surd \left(V\left(IC\right)\right)$	
EBGM	$EBGM=\left(a\left(a+b+c+d\right)\right)/\left(\left(a+c\right)\left(a+b\right)\right)$	EBGM05 > 2
	$95\%CI=e^{\ln\left(EBGM\right)\pm 1.96\sqrt{\left(\frac{1}{a}+\frac{1}{b}+\frac{1}{c}+\frac{1}{d}\right)}\;}$	

N, the number of reports; a is the number of cases where a specific adverse event occurred after using the target drug of the study, b is the number of cases where the target drug of the study were used but the specific adverse event did not occur, c is the number of cases where the specific adverse event occurred without the use of the target drug of the study, d is the number of cases where neither the target drug of the study were used nor the specific adverse event occurred; ROR, reporting odds ratio; γ, γij represent the parameters of the Dirichlet distribution; α, αi, β, βj represent the parameters of the Beta distribution; SD, standard deviation; ROR: reporting odds ratio, PRR: proportional reporting ratio, BCPNN: bayesian confidence propagation neural network, EBGM: empirical bayesian geometric mean; χ2, chi-squared; IC, information component; IC025, the lower limit of 95% CI, for the IC; E (IC), the IC, expectations; V(IC), the variance of IC; EEBGM05, the lower limit of the 95% CI, for EBGM; N: number of reports.

### 2.4 Time-to-onset analysis

First, we excluded records with (i) missing START_DT or EVENT_DT, (ii) EVENT_DT earlier than START_DT, (iii) implausible or non-parsable dates, and (iv) extreme intervals suggestive of data entry error. Subsequently, the time to onset of the event was calculated as the difference between the event date (EVENT_DT) and the drug start date (START_DT). The time to onset of different drug-related AEs was analyzed and compared, with the time interval described using the median and interquartile range.

### 2.5 Data visualization

The charts were generated using the ggplot2 package and GraphPad Prism (version 8.0.1). A world heat map was used to visualize the geographical distribution of submitted reports by country/region, and a line chart illustrated the annual number of adverse event reports from 2004 to 2024. Additionally, various scatter plots based on the study results were created to compare the incidence of AEs between the two drugs.

## 3 Result

### 3.1 Basic information of AEs

**FIGURE 1:**
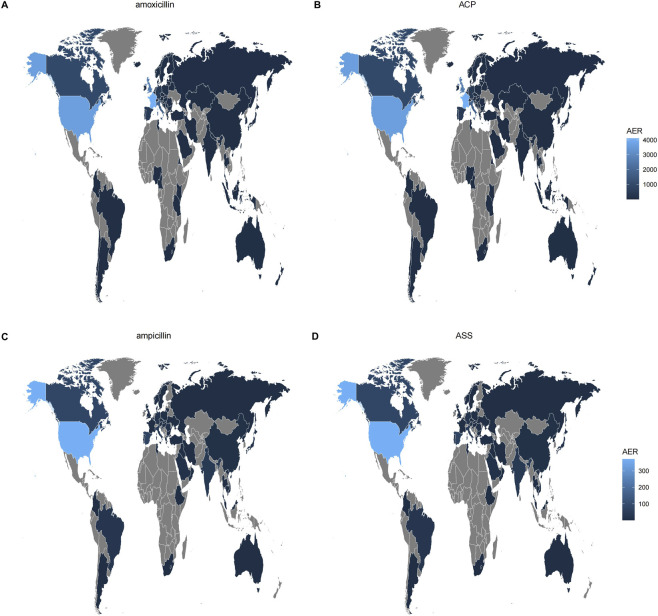
Global distribution of adverse event reporting rates for amoxicillin/amoxicillin-clavulanate, ampicillin/ampicillin-sulbactam.

**FIGURE 2:**
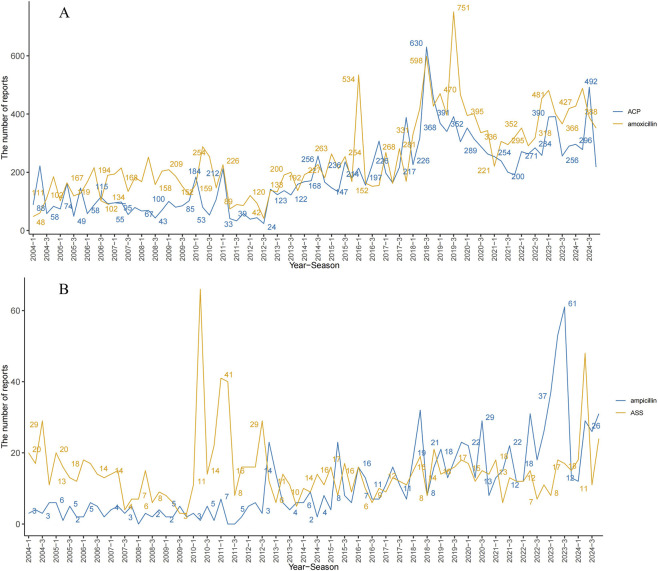
Trends in amoxicillin/amoxicillin-clavulanate, ampicillin/ampicillin-sulbactam adverse event reports (2004–2024).

This study analyzed the demographic characteristics and reporting patterns of AEs associated with four drug groups (Table 3). The total number of AE reports included for each group was as follows: 20,925 cases for the amoxicillin monotherapy group, 15,851 cases for the ACP group, 949 cases for the ampicillin monotherapy group, and 1,242 cases for the ASS group. Age distribution varied among the groups: the median age was 51 years (interquartile range [IQR]: 28–68) in the amoxicillin group, 56 years (34–71) in the ACP group, 39 years (21–65) in the ampicillin group, and 63 years (40–76) in the ASS group. Age stratification revealed a higher proportion of patients aged <60 years in the amoxicillin and ACP groups (46.76% and 42.61%, respectively), whereas the ASS group had the highest proportion of patients aged ≥60 years (45.49%). Regarding reporter type, consumers and physicians were the primary sources in the amoxicillin and ACP groups. In the ampicillin group, pharmacists submitted the highest proportion of reports (43.84%), while physicians were the main reporters in the ASS group (44.85%). In terms of adverse event outcomes, “other serious outcomes” and “hospitalization” were predominant across all groups. Notably, the ASS group had a relatively high proportion of fatal outcomes (9.22%), and the ampicillin group also reached 10.35%. The distribution of reporting countries (Figures 1A,B) showed that France, the United States, the United Kingdom, and Italy were the main contributors for the amoxicillin and ACP groups. For the ampicillin group, the United States, Canada, and Spain submitted more reports, whereas the ASS group primarily included reports from the United States, Japan, and Germany (Figures 1C,D). Regarding the route of administration, oral administration accounted for a relatively high proportion in the amoxicillin and ACP groups (50.32% and 43.71%, respectively), while intravenous administration was used in nearly half of the cases in the ASS group (49.92%). In terms of sex distribution, the amoxicillin group had a higher proportion of female patients (55.15%), whereas the ASS group had a slightly higher proportion of male patients (49.76%). The annual reporting trend indicated that the number of reports remained generally stable from 2018 to 2024 across all groups (Figure 2A). Specifically, the amoxicillin and ACP groups continued to show a high volume of reports in 2023–2024 (Figure 2B).

**TABLE 3:** Epidemiological characteristics of AE reports.

Characteristics	Amoxicillin Total	ACP Total
Age_yr	51.00 (28.00,68.00)	56.00 (34.00,71.00)
Age_yrQ		
<60	9,785 (46.76)	6,754 (42.61)
≥60	6,246 (29.85)	5,320 (33.56)
Unknow	4,894 (23.39)	3,777 (23.83)
Reporter		
Consumer	6,166 (29.47)	4,523 (28.53)
Physician	5,834 (27.88)	3,841 (24.23)
Other health-professional	4,191 (20.03)	3,304 (20.84)
Pharmacist	4,028 (19.25)	3,688 (23.27)
Unknown	682 (3.26)	484 (3.05)
Lawyer	18 (0.09)	9 (0.06)
Registered nurse	6 (0.03)	2 (0.01)
Outcomes		
Other serious	10,303 (48.45)	8,004 (47.79)
Hospitalization	8,013 (37.68)	6,078 (36.29)
Life threatening	1,659 (7.80)	1,381 (8.25)
Death	630 (2.96)	727 (4.34)
Disability	463 (2.18)	418 (2.50)
Required intervention to prevent permanent impairment/damage	136 (0.64)	108 (0.64)
Congenital anomaly	62 (0.29)	33 (0.20)
Reported countries		
France	4,104 (26.34)	698 (5.45)
Netherlands	256 (1.64)	211 (1.65)
Japan	140 (0.90)	NA
Poland	86 (0.55)	249 (1.94)
Russia	NA	197 (1.54)
Sweden	83 (0.53)	NA
Switzerland	NA	155 (1.21)
Belgium	71 (0.46)	68 (0.53)
Brazil	NA	138 (1.08)
Turkey	NA	117 (0.91)
Austria	NA	86 (0.67)
Croatia	NA	83 (0.65)
Greece	NA	75 (0.59)
United States	3,614 (23.19)	3,742 (29.20)
Czechia	NA	67 (0.52)
Australia	NA	56 (0.44)
Denmark	NA	56 (0.44)
United Kingdom	2,620 (16.81)	2,266 (17.68)
Italy	1749 (11.22)	1,242 (9.69)
Canada	967 (6.21)	467 (3.64)
Other	786 (5.04)	718 (5.60)
Spain	450 (2.89)	927 (7.23)
Germany	397 (2.55)	316 (2.47)
Portugal	259 (1.66)	882 (6.88)
Route		
Oral	10,510 (50.32)	6,896 (43.71)
Other	9,150 (43.81)	7,542 (47.80)
Intravenous	1,004 (4.81)	1,143 (7.24)
Transplacental	160 (0.77)	80 (0.51)
Intravenous drip	30 (0.14)	68 (0.43)
Intravenous bolus	12 (0.06)	38 (0.24)
Buccal	11 (0.05)	NA
Parenteral	NA	11 (0.07)
Intradermal	10 (0.05)	NA
Sex		
Female	11,540 (55.15)	8,069 (50.91)
Male	7,775 (37.16)	6,192 (39.06)
Unknown	1,610 (7.69)	1,590 (10.03)
TTO	2.00 (0.00,7.00)	2.00 (0.00,7.00)
TTOQ		
0–7	7,615 (57.63)	5,169 (57.11)
7–28	1992 (15.08)	1,396 (15.42)
28–60	389 (2.94)	304 (3.36)
≥60	191 (1.45)	118 (1.30)
Unknow	3,026 (22.90)	2064 (22.80)
Wt	68.04 (53.52,83.13)	71.00 (58.00,85.00)
Year		
2004	403 (1.93)	451 (2.85)
2005	512 (2.45)	430 (2.71)
2006	675 (3.23)	346 (2.18)
2007	724 (3.46)	322 (2.03)
2008	781 (3.73)	249 (1.57)
2009	669 (3.20)	362 (2.28)
2010	848 (4.05)	424 (2.67)
2011	475 (2.27)	344 (2.17)
2012	380 (1.82)	236 (1.49)
2013	661 (3.16)	541 (3.41)
2014	805 (3.85)	761 (4.80)
2015	898 (4.29)	696 (4.39)
2016	1,004 (4.80)	903 (5.70)
2017	880 (4.21)	966 (6.09)
2018	1774 (8.48)	1,629 (10.28)
2019	2083 (9.95)	1,404 (8.86)
2020	1,473 (7.04)	1,215 (7.67)
2021	1,143 (5.46)	885 (5.58)
2022	1,415 (6.76)	1,076 (6.79)
2023	1,667 (7.97)	1,327 (8.37)
2024	1,655 (7.91)	1,284 (8.10)

​	Ampicillin Total	ASS Total
Age_yr	39.00 (21.00,65.00)	63.00 (40.00,76.00)
Age_yrQ		
<60	359 (37.83)	464 (37.36)
≥60	169 (17.81)	565 (45.49)
Unknow	421 (44.36)	213 (17.15)
Reporter		
Pharmacist	416 (43.84)	368 (29.63)
Other health-professional	221 (23.29)	163 (13.12)
Physician	220 (23.18)	557 (44.85)
Consumer	72 (7.59)	100 (8.05)
Unknown	19 (2.00)	50 (4.03)
Registered nurse	1 (0.11)	​
Lawyer	​	4 (0.32)
Outcomes		
Other serious	540 (48.60)	692 (43.69)
Hospitalization	341 (30.69)	506 (31.94)
Life threatening	80 (7.20)	184 (11.62)
Death	115 (10.35)	146 (9.22)
Disability	6 (0.54)	22 (1.39)
Required intervention to prevent permanent impairment/damage	20 (1.80)	31 (1.96)
Congenital anomaly	9 (0.81)	3 (0.19)
Reported countries		
Other	428 (45.10)	762 (61.35)
United States	371 (39.09)	206 (16.59)
Canada	79 (8.32)	NA
Japan	NA	161 (12.96)
Spain	71 (7.48)	NA
Germany	NA	113 (9.10)
Route		
Other	594 (62.59)	490 (39.45)
Intravenous	206 (21.71)	620 (49.92)
Transplacental	121 (12.75)	​
Intravenous drip	​	70 (5.64)
Oral	28 (2.95)	49 (3.95)
Intramuscular	​	13 (1.05)
Sex		
Female	463 (48.79)	514 (41.38)
Male	306 (32.24)	618 (49.76)
Unknown	180 (18.97)	110 (8.86)
TTO	1.00 (0.00,5.00)	2.00 (0.00,6.00)
TTOQ		
0–7	77 (47.53)	432 (61.45)
7–28	18 (11.11)	95 (13.51)
28–60	2 (1.23)	17 (2.42)
≥60	2 (1.23)	5 (0.71)
Unknow	63 (38.89)	154 (21.91)
Wt	9.34 (1.97,77.00)	66.00 (50.00,83.98)
Year		
2004	16 (1.69)	77 (6.20)
2005	14 (1.48)	61 (4.91)
2006	15 (1.58)	62 (4.99)
2007	17 (1.79)	40 (3.22)
2008	9 (0.95)	37 (2.98)
2009	11 (1.16)	20 (1.61)
2010	10 (1.05)	113 (9.10)
2011	9 (0.95)	105 (8.45)
2012	31 (3.27)	73 (5.88)
2013	30 (3.16)	36 (2.90)
2014	25 (2.63)	44 (3.54)
2015	41 (4.32)	50 (4.03)
2016	43 (4.53)	42 (3.38)
2017	45 (4.74)	45 (3.62)
2018	75 (7.90)	63 (5.07)
2019	75 (7.90)	63 (5.07)
2020	72 (7.59)	58 (4.67)
2021	62 (6.53)	49 (3.95)
2022	87 (9.17)	45 (3.62)
2023	164 (17.28)	58 (4.67)
2024	98 (10.33)	101 (8.13)

### 3.2 Analysis by SOC level

All signal details are shown in Table 4. In the analysis at the SOC level, compared to the combination drug ACP, amoxicillin monotherapy showed stronger positive signals of association in Immune System Disorders (ROR = 6.23) and Skin and Subcutaneous Tissue Disorders (ROR = 4.08). In contrast, its combination formulation, ACP, exhibited a distinct risk profile. Although its associations with Immune System Disorders (ROR = 4.87) and Skin and Subcutaneous Tissue Disorders (ROR = 3.1) were slightly weaker, it demonstrated a markedly strong association signal for Hepatobiliary Disorders (ROR = 7.81 vs. ROR = 3.81 for amoxicillin). The ROR value was approximately twice that of amoxicillin monotherapy, highlighting the potential specific risk of liver injury introduced by the clavulanate component.

**TABLE 4:** Details of the SOC signals.

SOC	Amoxicillin	ROR (95% CI)	PRR (95% CI)	Chisq	IC(IC025) (95% CI)	EBGM(95% CI)	ACP	ROR (95% CI)	PRR (95% CI)	Chisq	IC(IC025) (95% CI)	EBGM(95% CI)
Immune system disorders	4,571	6.23 (6.05, 6.42)	5.88 (5.77, 6)	18,602.25	2.55 (2.5)	5.85 (5.7)	2,847	4.87 (4.69, 5.06)	4.67 (4.49, 4.86)	8,262.52	2.22 (2.16)	4.65 (4.51)
Skin and subcutaneous tissue disorders	13,135	4.08 (4, 4.16)	3.48 (3.41, 3.55)	24,524.81	1.8 (1.77)	3.47 (3.42)	8,205	3.1 (3.03, 3.17)	2.78 (2.73, 2.84)	9,843.57	1.47 (1.44)	2.77 (2.72)
Hepatobiliary disorders	2,379	3.81 (3.66, 3.97)	3.71 (3.57, 3.86)	4,736.5	1.89 (1.83)	3.7 (3.57)	3,674	7.81 (7.55, 8.08)	7.34 (7.06, 7.63)	20,173.74	2.87 (2.82)	7.3 (7.09)
Pregnancy, puerperium and perinatal conditions	587	1.96 (1.81, 2.13)	1.95 (1.8, 2.11)	273.94	0.96 (0.85)	1.95 (1.82)	231	0.98 (0.86, 1.12)	0.98 (0.85, 1.12)	0.09	−0.03 (-0.21)	0.98 (0.88)
Renal and urinary disorders	1915	1.5 (1.43, 1.57)	1.49 (1.43, 1.55)	310.43	0.57 (0.51)	1.49 (1.43)	1,184	1.18 (1.11, 1.25)	1.17 (1.1, 1.24)	30.91	0.23 (0.15)	1.17 (1.12)
Gastrointestinal disorders	8,280	1.43 (1.4, 1.47)	1.38 (1.35, 1.41)	949.95	0.46 (0.43)	1.38 (1.35)	7,425	1.67 (1.63, 1.72)	1.58 (1.55, 1.61)	1736.03	0.66 (0.63)	1.58 (1.55)
Blood and lymphatic system disorders	1,666	1.4 (1.33, 1.47)	1.39 (1.34, 1.45)	185.63	0.47 (0.4)	1.39 (1.33)	1,205	1.29 (1.22, 1.37)	1.28 (1.21, 1.36)	77.06	0.36 (0.28)	1.28 (1.22)
Ear and labyrinth disorders	376	1.25 (1.13, 1.38)	1.25 (1.13, 1.38)	18.23	0.32 (0.17)	1.24 (1.14)	207	0.87 (0.76, 1)	0.88 (0.77, 1.01)	3.69	−0.19 (-0.39)	0.88 (0.78)
Respiratory, thoracic and mediastinal disorders	4,088	1.23 (1.2, 1.27)	1.22 (1.17, 1.27)	170.84	0.29 (0.24)	1.22 (1.19)	3,243	1.25 (1.21, 1.3)	1.24 (1.19, 1.29)	154	0.31 (0.25)	1.24 (1.2)
Investigations	4,325	0.99 (0.96, 1.03)	1 (0.98, 1.02)	0.11	−0.01 (-0.05)	1 (0.97)	3,494	1.03 (0.99, 1.06)	1.03 (0.99, 1.07)	2.62	0.04 (-0.01)	1.03 (1)
Vascular disorders	1,473	0.97 (0.92, 1.02)	0.97 (0.91, 1.03)	1.17	−0.04 (-0.11)	0.97 (0.93)	1,158	0.98 (0.92, 1.03)	0.98 (0.92, 1.04)	0.67	−0.03 (-0.12)	0.98 (0.93)
Infections and infestations	2,958	0.79 (0.76, 0.82)	0.8 (0.77, 0.83)	160.21	−0.33 (-0.38)	0.8 (0.77)	3,033	1.05 (1.01, 1.09)	1.05 (1.01, 1.09)	6.22	0.06 (0.01)	1.04 (1.01)
Eye disorders	1,073	0.76 (0.71, 0.8)	0.76 (0.72, 0.81)	82.47	−0.39 (-0.48)	0.76 (0.72)	666	0.6 (0.55, 0.65)	0.6 (0.55, 0.65)	177.75	−0.73 (-0.84)	0.6 (0.57)
General disorders and administration site conditions	8,316	0.64 (0.62, 0.65)	0.68 (0.67, 0.69)	1,521.68	−0.56 (-0.59)	0.68 (0.67)	7,016	0.69 (0.68, 0.71)	0.73 (0.72, 0.74)	830.21	−0.45 (-0.48)	0.73 (0.72)
Metabolism and nutrition disorders	956	0.63 (0.59, 0.67)	0.63 (0.59, 0.67)	206.26	−0.66 (-0.75)	0.63 (0.6)	850	0.72 (0.67, 0.77)	0.72 (0.68, 0.76)	94.43	−0.47 (-0.57)	0.72 (0.68)
Nervous system disorders	3,761	0.61 (0.59, 0.63)	0.63 (0.61, 0.66)	876.56	−0.66 (-0.71)	0.63 (0.62)	2,665	0.55 (0.53, 0.57)	0.57 (0.55, 0.59)	929.6	−0.8 (-0.86)	0.57 (0.55)
Congenital, familial and genetic disorders	127	0.59 (0.49, 0.7)	0.59 (0.49, 0.7)	37.12	−0.77 (-1.02)	0.59 (0.51)	97	0.57 (0.47, 0.7)	0.57 (0.47, 0.69)	31.12	−0.8 (-1.09)	0.57 (0.48)
Cardiac disorders	1,053	0.56 (0.53, 0.59)	0.57 (0.54, 0.6)	361.65	−0.82 (-0.91)	0.57 (0.54)	1,170	0.8 (0.75, 0.85)	0.8 (0.75, 0.85)	58.43	−0.32 (-0.4)	0.8 (0.76)
Psychiatric disorders	2,207	0.54 (0.52, 0.56)	0.55 (0.53, 0.57)	844.66	−0.85 (-0.91)	0.55 (0.53)	1,270	0.39 (0.37, 0.41)	0.41 (0.39, 0.43)	1,167.3	−1.3 (-1.38)	0.41 (0.39)
Reproductive system and breast disorders	302	0.52 (0.47, 0.58)	0.52 (0.46, 0.58)	131.47	−0.93 (-1.09)	0.52 (0.48)	275	0.61 (0.54, 0.68)	0.61 (0.54, 0.69)	69.23	−0.71 (-0.88)	0.61 (0.55)
Musculoskeletal and connective tissue disorders	1,631	0.43 (0.41, 0.45)	0.44 (0.42, 0.46)	1,230.31	−1.18 (-1.26)	0.44 (0.42)	1,332	0.44 (0.42, 0.47)	0.46 (0.43, 0.49)	898.96	−1.12 (-1.2)	0.46 (0.44)
Injury, poisoning and procedural complications	2,684	0.38 (0.37, 0.4)	0.41 (0.39, 0.43)	2,587.97	−1.3 (-1.36)	0.41 (0.39)	1849	0.33 (0.32, 0.35)	0.36 (0.35, 0.37)	2,371.93	−1.49 (-1.55)	0.36 (0.34)
Endocrine disorders	42	0.23 (0.17, 0.32)	0.23 (0.17, 0.31)	105.24	−2.09 (-2.52)	0.23 (0.18)	29	0.21 (0.14, 0.3)	0.21 (0.14, 0.3)	88.45	−2.27 (-2.79)	0.21 (0.15)
Surgical and medical procedures	197	0.2 (0.18, 0.23)	0.21 (0.18, 0.24)	609.8	−2.28 (-2.48)	0.21 (0.18)	179	0.24 (0.2, 0.27)	0.24 (0.21, 0.28)	438.27	−2.06 (-2.27)	0.24 (0.21)
Neoplasms benign, malignant and unspecified (incl cysts and polyps)	137	0.07 (0.06, 0.09)	0.07 (0.06, 0.08)	1,635.33	−3.76 (-4)	0.07 (0.06)	125	0.08 (0.07, 0.1)	0.09 (0.08, 0.11)	1,246.49	−3.54 (-3.79)	0.09 (0.07)

SOC	Ampicillin	ROR (95% CI)	PRR (95% CI)	Chisq	IC(IC025) (95% CI)	EBGM(95% CI)	ASS	ROR (95% CI)	PRR (95% CI)	Chisq	IC(IC025) (95% CI)	EBGM(95% CI)
Pregnancy, puerperium and perinatal conditions	268	24.31 (21.44, 27.58)	22.06 (19.61, 24.81)	5,406.5	4.46 (4.28)	22.04 (19.83)	13	0.88 (0.51, 1.52)	0.88 (0.51, 1.52)	0.21	−0.18 (-0.94)	0.88 (0.56)
Immune system disorders	173	5.71 (4.89, 6.66)	5.41 (4.72, 6.21)	629.58	2.44 (2.22)	5.41 (4.76)	172	4.67 (4.01, 5.44)	4.48 (3.91, 5.14)	470.4	2.16 (1.94)	4.48 (3.94)
Congenital, familial and genetic disorders	32	3.7 (2.61, 5.24)	3.67 (2.58, 5.22)	62.25	1.87 (1.38)	3.67 (2.74)	​	​	​	​	​	​
Blood and lymphatic system disorders	163	3.49 (2.98, 4.09)	3.35 (2.86, 3.92)	272.96	1.74 (1.52)	3.35 (2.93)	122	2.11 (1.76, 2.53)	2.07 (1.74, 2.47)	68.93	1.05 (0.79)	2.07 (1.78)
Infections and infestations	302	2.12 (1.88, 2.39)	2 (1.81, 2.21)	158.99	1 (0.83)	2 (1.81)	266	1.5 (1.32, 1.7)	1.46 (1.3, 1.64)	40.96	0.55 (0.37)	1.46 (1.32)
Skin and subcutaneous tissue disorders	292	2 (1.77, 2.25)	1.89 (1.68, 2.13)	129.81	0.92 (0.75)	1.89 (1.71)	492	2.93 (2.67, 3.23)	2.65 (2.45, 2.87)	534.92	1.41 (1.27)	2.65 (2.45)
Renal and urinary disorders	94	1.82 (1.48, 2.24)	1.79 (1.47, 2.18)	33.71	0.84 (0.55)	1.79 (1.51)	93	1.48 (1.21, 1.82)	1.47 (1.21, 1.79)	14.21	0.56 (0.26)	1.47 (1.24)
Hepatobiliary disorders	42	1.64 (1.21, 2.22)	1.63 (1.21, 2.19)	10.3	0.7 (0.27)	1.63 (1.26)	135	4.41 (3.72, 5.24)	4.28 (3.66, 5.01)	341.93	2.1 (1.85)	4.27 (3.7)
Injury, poisoning and procedural complications	377	1.45 (1.3, 1.62)	1.39 (1.26, 1.53)	45.42	0.47 (0.32)	1.39 (1.27)	132	0.38 (0.32, 0.45)	0.41 (0.34, 0.49)	126.67	−1.3 (-1.55)	0.41 (0.35)
Metabolism and nutrition disorders	58	0.95 (0.73, 1.23)	0.95 (0.74, 1.23)	0.16	−0.08 (-0.45)	0.95 (0.76)	71	0.96 (0.76, 1.21)	0.96 (0.76, 1.21)	0.12	−0.06 (-0.4)	0.96 (0.79)
Respiratory, thoracic and mediastinal disorders	108	0.78 (0.65, 0.95)	0.79 (0.66, 0.94)	6.16	−0.34 (-0.61)	0.79 (0.67)	206	1.27 (1.1, 1.46)	1.25 (1.09, 1.43)	10.99	0.32 (0.12)	1.25 (1.11)
Vascular disorders	48	0.78 (0.59, 1.04)	0.78 (0.59, 1.03)	2.91	−0.35 (-0.76)	0.78 (0.62)	101	1.37 (1.12, 1.67)	1.36 (1.12, 1.65)	9.74	0.44 (0.16)	1.36 (1.15)
Investigations	125	0.69 (0.58, 0.83)	0.71 (0.6, 0.85)	15.98	−0.5 (-0.75)	0.71 (0.61)	408	2.04 (1.84, 2.26)	1.91 (1.73, 2.11)	189.5	0.93 (0.79)	1.91 (1.75)
Cardiac disorders	51	0.67 (0.51, 0.89)	0.68 (0.52, 0.89)	7.91	−0.56 (-0.95)	0.68 (0.54)	137	1.52 (1.28, 1.8)	1.5 (1.28, 1.75)	23.36	0.58 (0.34)	1.5 (1.3)
General disorders and administration site conditions	330	0.62 (0.55, 0.69)	0.66 (0.6, 0.73)	69.6	−0.6 (-0.76)	0.66 (0.6)	401	0.62 (0.56, 0.69)	0.67 (0.61, 0.74)	80.48	−0.58 (-0.73)	0.67 (0.61)
Surgical and medical procedures	21	0.54 (0.35, 0.82)	0.54 (0.35, 0.83)	8.39	−0.89 (-1.5)	0.54 (0.38)	16	0.34 (0.21, 0.55)	0.34 (0.21, 0.55)	20.59	−1.55 (-2.24)	0.34 (0.23)
Gastrointestinal disorders	111	0.43 (0.36, 0.52)	0.46 (0.39, 0.55)	79.3	−1.14 (-1.41)	0.46 (0.39)	261	0.88 (0.77, 0.99)	0.89 (0.79, 1)	4.23	−0.18 (-0.36)	0.89 (0.8)
Eye disorders	24	0.41 (0.28, 0.62)	0.42 (0.28, 0.62)	19.76	−1.26 (-1.82)	0.42 (0.3)	23	0.33 (0.22, 0.49)	0.33 (0.22, 0.5)	31.55	−1.59 (-2.17)	0.33 (0.24)
Nervous system disorders	90	0.36 (0.29, 0.44)	0.38 (0.31, 0.46)	101.97	−1.41 (-1.71)	0.38 (0.32)	164	0.54 (0.46, 0.63)	0.56 (0.48, 0.66)	61.23	−0.83 (-1.06)	0.56 (0.49)
Reproductive system and breast disorders	7	0.3 (0.14, 0.64)	0.3 (0.14, 0.63)	11.2	−1.71 (-2.72)	0.3 (0.16)	​	​	​	​	​	​
Psychiatric disorders	27	0.16 (0.11, 0.23)	0.17 (0.12, 0.25)	118.05	−2.57 (-3.11)	0.17 (0.12)	73	0.36 (0.28, 0.45)	0.37 (0.29, 0.47)	81.77	−1.42 (-1.76)	0.37 (0.31)
Musculoskeletal and connective tissue disorders	25	0.16 (0.11, 0.23)	0.17 (0.11, 0.25)	111.12	−2.59 (-3.15)	0.17 (0.12)	42	0.22 (0.16, 0.3)	0.23 (0.17, 0.31)	113.89	−2.12 (-2.55)	0.23 (0.18)
Ear and labyrinth disorders	​	​	​	​	​	​	9	0.61 (0.31, 1.17)	0.61 (0.32, 1.16)	2.3	−0.72 (-1.62)	0.61 (0.35)
Endocrine disorders	​	​	​	​	​	​	4	0.45 (0.17, 1.21)	0.46 (0.17, 1.23)	2.62	−1.14 (-2.4)	0.46 (0.2)
Neoplasms benign, malignant and unspecified (incl cysts and polyps)	​	​	​	​	​	​	5	0.05 (0.02, 0.13)	0.05 (0.02, 0.12)	83.53	−4.19 (-5.34)	0.05 (0.03)

The most prominent positive signal for ampicillin monotherapy was concentrated in Pregnancy, puerperium and perinatal conditions, with a remarkably high ROR of 24.31, indicating an extremely strong association. Clear positive signals were also detected for Immune system disorders (ROR = 5.71), Various congenital, familial and genetic disorders (ROR = 3.70), and Blood and lymphatic system disorders (ROR = 3.49). In contrast, the signal strength for ASS in Hepatobiliary disorders (ROR = 4.41) far exceeded that of ampicillin monotherapy (ROR = 1.64). For Skin and subcutaneous tissue disorders, the risk signal for ASS (ROR = 2.93) was also higher than that for the monotherapy (ROR = 2.00). Furthermore, ASS demonstrated a unique signal in Investigations (ROR = 2.04), whereas the monotherapy showed a negative signal in this category.

### 3.3 Analysis by PT level

**FIGURE 3:**
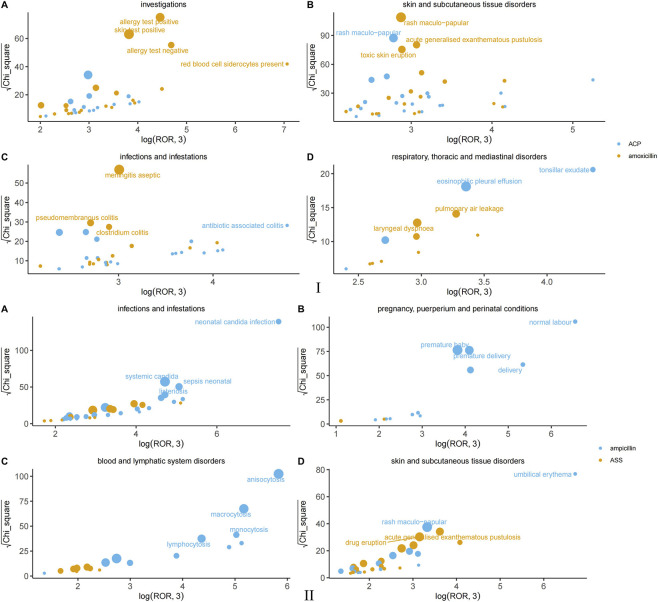
Comparison of adverse event signals by organ system: amoxicillin/amoxicillin-clavulanate, ampicillin/ampicillin-sulbactam.

All signal details are shown in Supplementary Materials 1, 2. At the PT-level analysis, the monotherapy and combination drugs similarly exhibited distinct adverse reaction profiles (Figure 3). In Skin and subcutaneous tissue disorders, amoxicillin showed stronger signals for Urticaria (ROR 8.57 vs. ACP 5.74), Angioedema (ROR 13.71 vs. 7.37), and Maculopapular rash (ROR 23.5 vs. 21.21). In contrast, ACP had stronger signals for Pruritic rash (ROR 5.35 vs. 5.61) and Fixed rash (ROR 11.83 vs. 19.88). In Hepatobiliary disorders, the signals for ACP were far higher than those for amoxicillin for Drug-induced liver injury (ROR 35.41 vs. 4.98), Cholestatic hepatitis (ROR 43.41 vs. 21.82), and Cholestatic jaundice (ROR 37.49 vs. 16.77). Regarding the Immune system, amoxicillin had more prominent signals for Anaphylactic shock (ROR 26.61 vs. 17.26) and Type I hypersensitivity (ROR 57.11 vs. 32.2), while ACP was slightly higher for Type IV hypersensitivity (ROR 53.32 vs. 45.31). Additionally, ACP showed stronger signals for renal events such as Crystalluria (ROR 21.23 vs. 180.48) and Chromaturia (ROR 12.17 vs. 5.57), whereas amoxicillin was more significant for local reactions like Lip edema (ROR 44.67 vs. 34.55) and Face edema (ROR 20.46 vs. 15.91).

Ampicillin monotherapy and its combination drug ASS demonstrated significantly different adverse reaction signal characteristics. The most prominent positive signals for ampicillin monotherapy were concentrated in Pregnancy and neonatal-related events: Premature baby (ROR = 66.65), Premature delivery (ROR = 90.05), and Low birth weight baby (ROR = 92.65, case count = 35) all showed extremely strong associations. The signal strengths for Normal newborn (ROR = 1,340.79) and Neonatal *candida* infection (ROR = 3,910.25) were particularly notable. 1t must be emphasized that “Normal newborn” is merely a reported event term and a strong association signal, rather than a pathological AE caused by ampicillin. In the Blood system, strong signals were observed for Anisocytosis (ROR = 606.87), Macrocytosis (ROR = 292.37), and Monocytosis (ROR = 249.7). Within the Skin system, clear associations were found for Maculopapular rash (ROR = 38.65) and Toxic epidermal necrolysis (ROR = 24.73). In contrast, ASS exhibited a unique and stronger risk profile. Its signal for Hemorrhagic enterocolitis in Gastrointestinal disorders was exceptionally strong (ROR = 580.56), far exceeding that of ampicillin monotherapy (ROR = 68.55). For Skin adverse reactions, the signals for Acute generalized exanthematous pustulosis (ROR = 53.29), Linear IgA disease (ROR = 88.31), and Drug eruption (ROR = 31.92) were all significantly stronger than those for the monotherapy. Regarding Allergic and immune reactions, ASS showed prominent signals for Anaphylactic shock (ROR = 25.78) and Allergic coronary spasm syndrome (ROR = 100.41). Furthermore, ASS demonstrated widespread positive signals in Investigations, such as Stool culture positive (ROR = 697.42) and *Klebsiella* test positive (ROR = 270.72). Other infection-related PTs, including Clostridioides difficile colitis (ROR = 67.46), were also detected, suggesting a closer association with intestinal flora disturbance and specific pathogen infections.

### 3.4 Subgroup analysis

**FIGURE 4:**
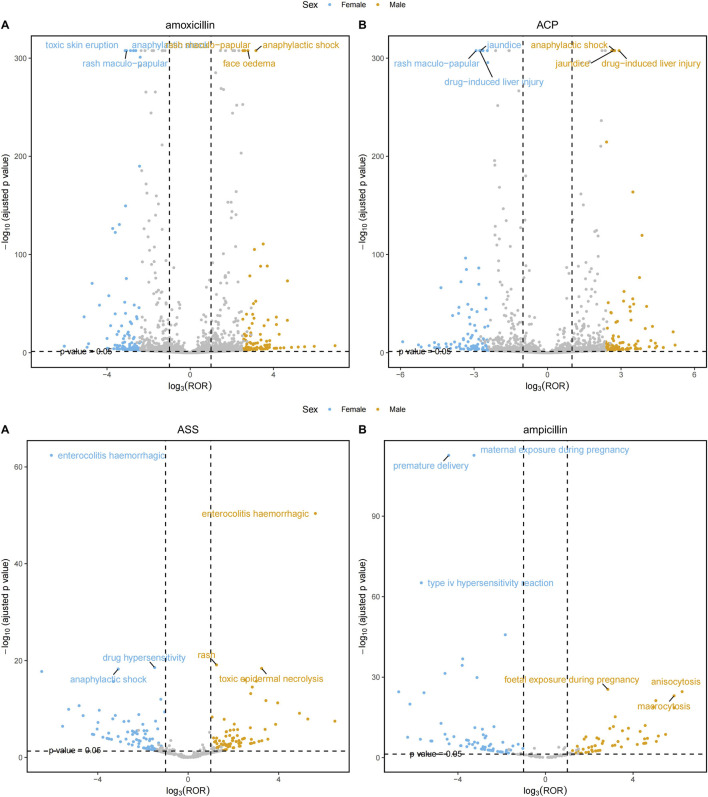
Adverse event risks stratified by gender: amoxicillin/amoxicillin-clavulanate, ampicillin/ampicillin-sulbactam.

Subgroup analyses revealed that the identified safety signals were modified by age and gender (Figure 4). All signal details are shown in Supplementary Materials3–6.

#### 3.4.1 Grouped by sex

In female patients, amoxicillin monotherapy was associated with strong signals for multiple adverse reactions. Particularly within Skin and subcutaneous tissue disorders, signals were notably high for scarlatiniform rash (ROR = 80.86), prurigo (ROR = 37.42), and acute generalized exanthematous pustulosis (ROR = 30.42). Furthermore, it showed strong associations for Immune system reactions (e.g., Type I hypersensitivity, ROR = 52.30) and Gastrointestinal system reactions (e.g., lip edema, ROR = 42.13). In contrast, when female patients used ACP, the signal strengths for the same categories of adverse reactions were generally attenuated. For instance, the ROR for scarlatiniform rash decreased to 52.45, and for Type I hypersensitivity to 30.06. This indicates that for female patients, the risk signals for inducing various adverse reactions—especially skin and hypersensitivity reactions—are significantly higher with amoxicillin monotherapy than with ACP. In male patients, the risk pattern differed. Certain skin-related adverse reaction signals induced by amoxicillin monotherapy were even stronger than those in females. For example, the ROR for scarlatiniform rash was as high as 109.61, and for symmetrical drug-related intertriginous and flexural exanthema (SDRIFE), it was 40.63. Additionally, the risk for anaphylactic shock (ROR = 32.16) was higher than that observed in females (ROR = 23.45). When male patients used ACP, although signals for skin reactions were relatively weaker, stronger and more specific signals for hepatobiliary and renal system injuries emerged. For instance, the RORs for cholestatic hepatitis (45.89), choluria (110.34), and crystalluria (169.96) were all significantly higher than their corresponding values in female patients.

In female patients, ampicillin monotherapy demonstrated exceptionally high risk signals for skin and subcutaneous tissue disorders: Maculopapular rash (ROR = 63.8), Acute generalized exanthematous pustulosis (ROR = 29.9), and Toxic epidermal necrolysis (ROR = 34.6). All these signal strengths were substantially higher than those for ASS-related events (with corresponding RORs of 15.0 and 43.7 for maculopapular rash and acute generalized exanthematous pustulosis, respectively, and no significant signal detected for toxic epidermal necrolysis). At the immune system level, ampicillin triggered an exceptionally strong signal for Type IV hypersensitivity (ROR = 507.5), whereas no significant signal was detected for ASS in this category. This suggests a markedly increased combined risk of severe skin and hypersensitivity reactions when female patients use ampicillin monotherapy. In male patients, compared to ASS, ampicillin monotherapy still showed relatively prominent signals in skin and subcutaneous tissue disorders, such as Maculopapular rash (ROR = 25.3) and Drug reaction with eosinophilia and systemic symptoms (DRESS) (ROR = 12.0). Although the absolute values were slightly lower than those observed in females, they remained higher than the signals for ASS in males (maculopapular rash ROR = 9.1; no significant signal detected for DRESS). However, ASS demonstrated strong signals in the renal/urinary system of male patients, for example, Crystalluria (ROR = 314.8) and Tubulointerstitial nephritis (ROR = 30.3), with values significantly higher than those observed in female patients.

#### 3.4.2 Grouped by age

Amoxicillin and its combination drug (ACP) exhibited a “bipolar” characteristic in their age-stratified adverse event spectra. The younger-than-60-year group was primarily associated with skin and Type I hypersensitivity reactions, whereas the 60-year-or-older group showed a shift toward Clostridioides difficile infection (CDI) and hepatobiliary/renal toxicities. Specifically, in the <60 years population, the strongest signal for amoxicillin was for Acute generalized exanthematous pustulosis (ROR 26.1, 95% CI 22.0–31.0). The associated Type I hypersensitivity (ROR 45.6) and Urticaria (ROR 8.0) were both significantly higher than in the older group. Conversely, in the ≥60 years group, the risk for Clostridioides difficile colitis increased sharply, with an ROR of 13.9 for amoxicillin (compared to only 8.6 in the <60 years group) and rising further to 28.6 for ACP, suggesting greater vulnerability of the intestinal microbial barrier in the elderly. Similarly, hepatobiliary toxicity was prominent only in the older age group: the ROR for Drug-induced liver injury associated with amoxicillin was 14.8 in the ≥60 years group, approximately four times that in the <60 years group (3.9). For ACP, the ROR for Cholestatic hepatitis reached 24.7 in the ≥60 years group, significantly higher than the corresponding value in the younger group (17.0). Regarding renal function, the signal for Crystalluria in the ACP cohort was markedly elevated in the ≥60 years group (ROR 310), compared to only 33.6 in the <60 years group, accompanied by a concurrent increase in Tubulointerstitial nephritis (ROR 9.4 vs. 3.4).

In contrast to the amoxicillin groups, the safety risks associated with ampicillin monotherapy increased with age primarily through a “resistance-infection” cascade, whereas ASS manifested a distinct “eosinophil-hematologic axis” toxicity specific to the elderly population. Firstly, concerning signals for pathogen resistance, in the <60 years population, the signal strength associated with ampicillin (ROR 28.15) was already significantly higher than that for ASS. This signal increased sharply by 2.6-fold (ROR 74.89) in the ≥60 years group, indicating a greater risk of inducing resistance with ampicillin use in the elderly. Conversely, ASS did not show significant signals in the same resistance category across age groups. Secondly, hematologic toxicity exhibited a contrasting age-related pattern. Ampicillin-associated thrombocytopenia showed only a mild elevation in the <60 years group (ROR 4.43), with no further increase in the ≥60 years group. In stark contrast, ASS elicited a strong signal for eosinophilia even in the <60 years group (ROR 14.21). This signal intensity increased nearly fourfold in the ≥60 years group (ROR 53.79), accompanied by severe hematologic events such as neonatal thrombocytopenia and monocytosis, which appeared exclusively in the elderly population.

### 3.5 The median time to onset

**FIGURE 5:**
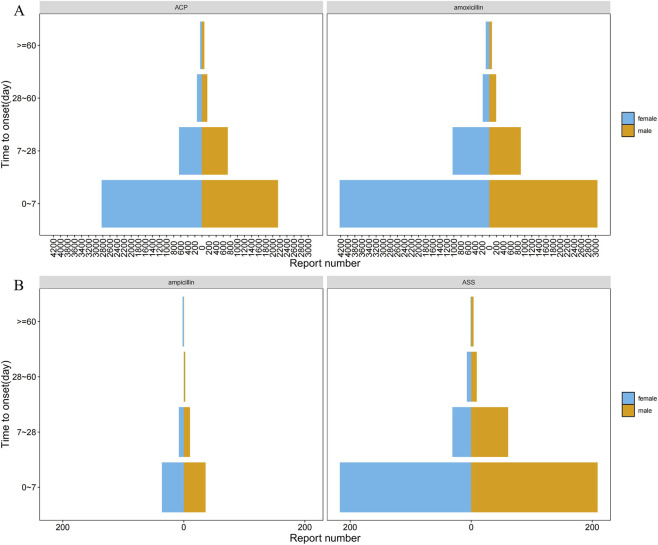
Gender distribution in time-to-onset of adverse events: amoxicillin/amoxicillin-clavulanate, ampicillin/ampicillin-sulbactam.

The median time to onset of AEs was 2 days for both the amoxicillin and ACP groups (Figure 5A), and 1 day and 2 days for the ampicillin and ASS groups (Figure 5B), respectively. The majority of events occurred within the first 7 days after drug administration. After this period, the number of events in males declined rapidly, whereas the decline in females was markedly delayed. This pattern suggests that females exhibit a “rapid-onset, delayed-resolution” biphasic characteristic in response to both drugs. Consequently, extending the clinical monitoring period for female patients receiving these medications to 4 weeks may be warranted.

## 4 Discussion

This study, utilizing over 200,000 reports from the FAERS database spanning 2004 to 2024, represents the first large-sample, real-world systematic comparison of the changes in the safety profiles of penicillin antibiotics before and after the addition of BLIs. The results demonstrate that the introduction of **β**-lactamase inhibitors significantly alters the adverse event characteristics of the parent antibiotics. This finding underscores the necessity for personalized monitoring and management in clinical practice, tailored to the specific risks associated with different combination formulations.

First, this study found distinct differences in the adverse event profiles between amoxicillin monotherapy and ACP. Amoxicillin monotherapy showed relatively stronger signals in immune system disorders (such as anaphylactic shock and Type I hypersensitivity) and skin reactions (such as urticaria and angioedema), with its system-level ROR values slightly higher than those of ACP. This suggests that the occurrence of allergic reactions in patients taking ACP is not significantly different from that observed with amoxicillin alone. Previous research has reported positive basophil activation tests to amoxicillin (AX), clavulanic acid (CLV), and AX-CLV in immediate allergic reactions to ACP, whereas patients with non-immediate reactions exhibited dendritic cell and T-lymphocyte responses specific to AX and CLV. That study therefore suggested that allergic reactions to both AX and CLV may co-occur in patients allergic to ACP (Salas et al., 2017). Another study similarly detected specific T-cell clones responsive to amoxicillin and clavulanic acid, respectively, in patients with immediate allergic reactions to ACP, with proliferative responses being dose-dependent and no cross-reactivity observed (Ariza et al., 2020). Consequently, some researchers have proposed that clavulanic acid is the primary culprit in immediate allergic reactions to penicillins among **β**-lactams and may increase the allergic risk of ACP (Torres-Rojas et al., 2023). However, due to the small sample sizes of these studies, this theory remains controversial. A large-scale population-based study did not observe a difference in allergy issues between amoxicillin alone and ACP (Savage et al., 2023). Our results are consistent with the findings of that population study, likewise finding no significant difference between the two. This may help clarify the common clinical concern that enzyme inhibitor-containing combination formulations (like ACP) might additionally increase the risk of allergy. Our findings indicate that, compared to amoxicillin alone, ACP does not significantly elevate the overall risk of allergic reactions. This suggests to clinicians that when deciding between amoxicillin and its combination formulation ACP, allergy prevention may not be a critical factor in the decision-making process.

In contrast, ACP demonstrated a significantly higher risk for hepatobiliary system disorders, particularly drug-induced liver injury and cholestatic hepatitis, with its ROR values approximately twice those of amoxicillin. This finding is consistent with several previous studies. A United Kingdom retrospective study reported incidence rates of acute liver injury per 10,000 prescriptions for ACP and amoxicillin alone as 1.7 (95% CI 1.1–2.7) and 0.3 (0.2–0.5), respectively. Furthermore, the risk of acute liver injury was more than three times higher following a second or subsequent course of ACP compared to a single course (García Rodríguez et al., 1996). Additionally, two population-based studies on drug-induced liver injury found a higher risk of acute liver injury with ACP compared to amoxicillin alone (Ortland et al., 2021; Ammendolia et al., 2025). Consequently, some researchers recommend cautious use of ACP in patients with hepatic dysfunction and advise against its use in those with a history of cholestatic jaundice/hepatic dysfunction (White et al., 2004).

The underlying mechanisms for this phenomenon are not yet fully understood. A recent retrospective study analyzing data from the global pharmacovigilance database VigiBase identified the amoxicillin/clavulanate combination as one of the most frequently suspected drugs in serious drug-related liver disorders. The authors concluded that the cause of ACP-induced hepatotoxicity remains unclear and postulated an immunoallergic mechanism (de Abajo et al., 2004). Research has demonstrated that ACP induces hepatitis by triggering the infiltration of immune factors associated with immunoallergic injury and various T cells (predominantly CD8^+^ cytotoxic T cells) into the portal triads (Foureau et al., 2014). These findings suggest that the pathogenesis of the liver injury may be primarily an immune-mediated attack on cholangiocytes or perhaps on the apical pole of hepatocytes. Currently, the specific antigens driving this cellular immune response are unknown, but they are likely neoantigens generated from the **β**-lactam structures of ACP, which may react with proteins in susceptible hosts (deLemos et al., 2016). Although many individuals taking ACP may form neoantigens, drug-induced liver injury (DILI) likely manifests only in a small subgroup with specific genetic predispositions. Several studies have proposed that class I and II HLA genotypes influence susceptibility to amoxicillin/clavulanate-induced liver injury, underscoring the importance of the adaptive immune response in ACP-related hepatotoxicity (Lucena et al., 2011). Recent analysis of genotype-phenotype interactions in ACP-DILI cases found that class I alleles A3002 and B1801 were more frequently associated with hepatocellular injury compared to controls, while the DRB1*1501-DQB1*0602 haplotype was significantly increased in cholestatic/mixed cases (Stephens et al., 2013). Further research is needed to elucidate the related mechanisms.

Second, the signals for ampicillin monotherapy were primarily concentrated in pregnancy and puerperium events and hematologic system abnormalities, with its related system-level ROR values far exceeding those of ASS, which may be related to its favorable placental penetration, extensive use during pregnancy, and neonatal exposure. Ampicillin readily crosses the placental barrier, achieving effective concentrations in amniotic fluid and fetal blood. This property makes it a frequent choice for treating infections during pregnancy, Ampicillin monotherapy has traditionally been commonly used for the prevention of Group B *Streptococcus* (GBS) and puerperal infections in the perinatal period (Stephens et al., 2013; McDuffie et al., 1996; Ziogos et al., 2010). For instance, listeriosis is a rare but serious infectious disease during pregnancy that can have devastating consequences for the fetus and newborn. A high-quality meta-analysis indicates that intravenous ampicillin is the first-line treatment regimen (Khsi et al., 2022). Chorioamnionitis is the most common infection-related diagnosis in the delivery room and a precursor to puerperal infection and neonatal sepsis. Professional society guidelines recommend ampicillin combined with gentamicin as the first-line antimicrobial regimen (Conde-Agudelo et al., 2020). Additionally, early-onset sepsis (EOS) remains a significant cause of neonatal morbidity and mortality. When EOS is suspected, ampicillin and gentamicin constitute the appropriate empiric regimen in most cases (Flannery et al., 2025).

However, its frequent use in pregnancy also directly more exposes the fetus to the drug. This exposure may lead to direct fetal effects (such as impacts on the hematopoietic system) or induce uterine contractions, thereby increasing the risk of adverse pregnancy outcomes like prematurity, neonatal infection, and low birth weight. A study involving patients with PROM at 25–35 weeks of gestation found that compared to ASS, the use of ampicillin monotherapy was associated with a significantly higher number of neonatal complications (20 vs. 5, P < 0.001) (L et al., 1995). Another head-to-head comparative drug study found that in pre-cesarean section prophylaxis, ampicillin monotherapy was associated with a higher incidence of postoperative endometritis (35.3% vs. 8.8%, p < 0.02) (Rijhsinghani et al., 1995). These findings are consistent with our results, indicating that for perinatal patients, the common misconception that “combination drugs inherently carry higher risks due to more components” should be corrected when considering ASS, and attention should instead be paid to its actual risk profile in this specific population. Interestingly, ampicillin and sulbactam are two independent molecules in the bloodstream. Both can cross the placenta, so theoretically, with the combination drug (ASS), the fetus faces “dual exposure” to both ampicillin and sulbactam. Therefore, the fact that the signal strength for ASS at the perinatal level not only does not increase but shows a sharp decline in ROR is perplexing.

In contrast, ASS demonstrates stronger signals in gastrointestinal disorders (e.g., hemorrhagic enterocolitis), skin adverse reactions (e.g., acute generalized exanthematous pustulosis), and allergic reactions (e.g., Kounis syndrome). This suggests that the inclusion of sulbactam not only restores antibacterial activity but may also fundamentally alter the safety profile of this combination product through its distinct chemical and immunological properties. Nevertheless, the precise mechanisms underlying these observations remain unclear. Further research may be needed to explain the underlying mechanisms behind this observation to improve our understanding.

The subgroup analysis further revealed gender- and age-specific differences in the safety profiles of amoxicillin versus ACP. Regarding gender, female patients using amoxicillin monotherapy showed significantly stronger risk signals for skin/subcutaneous tissue disorders (e.g., scarlatiniform rash, prurigo) and Type I hypersensitivity compared to using ACP, suggesting that amoxicillin alone may be more likely to induce immune reactions characterized by immediate allergy and skin manifestations in females. In contrast, male patients using ACP, while having relatively weaker skin reaction signals, exhibited significantly elevated risk signals for hepatobiliary (e.g., cholestatic hepatitis) and renal system injuries (e.g., crystalluria), which were markedly higher than corresponding values in females. This gender-divergent pattern suggests that the introduction of clavulanate may preferentially direct toxicity toward liver and kidney pathways in males, possibly via gender-specific pharmacokinetic or immune-response mechanisms. Age stratification showed a clear dichotomy in adverse event spectra: patients aged <60 years primarily faced risks centered on skin and immediate hypersensitivity reactions, whereas those ≥60 years were more susceptible to complications like Clostridioides difficile infection, drug-induced liver injury, and crystalluria. This shift is likely related to age-associated immunosenescence, decreased stability of gut microbiota, and declining hepatic/renal metabolic function. Clinically, these findings suggest the need for individualized risk assessment and monitoring: for younger, especially female patients, vigilance for severe skin allergies is warranted with amoxicillin monotherapy; for elderly, especially male patients, using ACP requires focused monitoring of liver function and urine crystals, along with awareness of secondary intestinal infections.

Similarly, in the comparison between ampicillin and ASS, the subgroup analysis highlighted distinct risk features with significant population heterogeneity. Gender analysis indicated that female patients using ampicillin monotherapy exhibited exceptionally strong risk signals for skin toxicity (e.g., maculopapular rash, toxic epidermal necrolysis) and Type IV delayed hypersensitivity, far exceeding those for ASS. This suggests ampicillin may more readily form highly immunogenic drug-protein complexes in females, triggering intense T-cell-mediated responses. However, male patients using ASS, while having relatively lower skin reaction risks, showed notable renal/urinary system toxicity signals (e.g., crystalluria, tubulointerstitial nephritis) significantly stronger than in females. This implies that the addition of sulbactam may induce specific renal injury risks in males, potentially via gender-dependent renal excretion. Age-stratified analysis further revealed evolving risk patterns: ampicillin monotherapy showed sharply increased signals related to pathogen resistance in elderly patients (≥60 years), suggesting it may more readily drive resistant bacteria selection and secondary infections in this population. Conversely, ASS triggered a unique “eosinophil-hematologic axis” toxicity in the elderly, manifesting as strong eosinophilia and associated severe hematologic events. These findings of age and gender interactions not only underscore the limitations of a “one-size-fits-all” safety assessment model but also suggest that clinical decision-making for ampicillin or ASS in perinatal, elderly, and different gender groups requires differentiated risk-benefit weighing and monitoring strategies. For instance, renal function and complete blood counts should be closely monitored when ASS is used in elderly males, whereas heightened vigilance for severe skin adverse reactions is crucial when ampicillin monotherapy is prescribed to women of childbearing age.

However, this study also has several limitations. Firstly, as a spontaneous reporting system, FAERS inherently suffers from known biases such as under-reporting, stimulated reporting, differential reporting by country and reporter type, duplicate reporting, missingness in key covariates, which may affect the accuracy and representativeness of the AE signals. Additionally, due to the relatively small number of events, we acknowledge reduced power for rare events in the ampicillin cohort and interpret extreme signals with caution. Secondly, this study did not adequately control for potential confounding factors, including patients’ underlying conditions, concomitant medications, drug doses, and treatment duration. This may interfere with the interpretation of some association signals. However, this study mitigates the influence of confounding factors to some extent through multiple algorithmic cross-validation, and subgroup analysis. Finally, this study focused solely on two combination agents—ACP and ASS—and did not include other widely used BLI combinations (e.g., piperacillin-tazobactam). Therefore, the comprehensiveness of the conclusions needs to be expanded. Future research can be linked with health databases (e.g., electronic health record (EHR) data) to obtain more detailed clinical information, and then use methods such as propensity score matching and multivariate regression models to control for confounding factors. Additionally, multiple databases (e.g., VigiBase, EudraVigilance) can be leveraged for cross-validation to enhance the accuracy of similar studies.

## 5 Conclusion

The addition of β-lactamase inhibitors may alter the adverse event profiles of parent antibiotics. In clinical practice, individualized risk assessment and monitoring should be implemented based on patient demographic characteristics. Further studies are needed to investigate whether similar phenomena occur with other combination formulations, to better understand and optimize the use of β-lactam antibiotics.

## Data Availability

The original contributions presented in the study are included in the article/Supplementary Material, further inquiries can be directed to the corresponding author.
